# Visual search performance in ‘CCTV’ and mobile phone-like video footage

**DOI:** 10.1186/s41235-021-00326-w

**Published:** 2021-09-24

**Authors:** Viktoria R. Mileva, Peter J. B. Hancock, Stephen R. H. Langton

**Affiliations:** Psychology, Faculty of Natural Sciences, University of Stirling, Cottrell Building, Stirling, FK9 4LA UK

## Abstract

Finding an unfamiliar person in a crowd of others is an integral task for police officers, CCTV-operators, and security staff who may be looking for a suspect or missing person; however, research suggests that it is difficult and accuracy in such tasks is low. In two real-world visual-search experiments, we examined whether being provided with four images versus one image of an unfamiliar target person would help improve accuracy when searching for that person through video footage. In Experiment 1, videos were taken from above and at a distance to simulate CCTV, and images of the target showed their face and torso. In Experiment 2, videos were taken from approximately shoulder height, such as one would expect from body-camera or mobile phone recordings, and target images included only the face. Our findings suggest that having four images as exemplars leads to higher accuracy in the visual search tasks, but this only reached significance in Experiment 2. There also appears to be a conservative bias whereby participants are more likely to respond that the target is not in the video when presented with only one image as opposed to 4. These results point to there being an advantage for providing multiple images of targets for use in video visual-search.

## Significance statement

In two experiments we show that when looking for an individual from CCTV or phone-camera- like footage, participants are more accurate in deciding whether the individual is there or not when they have studied four images of the individual rather than one. Previous research has shown similar results using static faces. Our results extend this work to a situation where participants search through real-world video footage, a task typically faced by CCTV-operators, security guards, and police officers when looking for missing persons or suspects.

## Introduction

If you had witnessed a crime in a city centre in the 1980’s, you may have been asked by police to recall, from memory, the appearance of the perpetrator, in order for a professional sketch artist to draw a likeness. This composite sketch would then be distributed to local police stations and media outlets. Thirty years later, there are CCTV cameras surveilling most city centres, which provide real-time tracking of events in high definition. In addition, there are 3.2 billion smartphone users worldwide (O’Dea, 2020), any one of whom can record videos or capture images of an event at various distances and qualities. These technologies can be used to help law enforcement and families, by providing footage or images of perpetrators and missing persons, instead of sketches created from memory. Indeed, the FBI have recently released compilation videos and images taken from CCTV and mobile phone devices of culprits in the January 6th 2021 Capitol Riots in the USA (https://www.fbi.gov/wanted/capitol-violence), in a bid to help identify the intruders.

In previous literature using visual search or face matching paradigms, familiarity has been shown to play a key role in accuracy. That is, we are better able to recognise a target individual who is familiar to us even when lighting, pose, and expression vary (Burton et al., 2015); however, these same parameters hinder our ability to recognise unfamiliar faces (Hancock et al., 2000). Indeed, familiar and unfamiliar faces are thought to be processed differently (Johnston & Edmonds, 2009; Natu & O’Toole, 2011), and we are much more accurate when picking familiar individuals out from CCTV footage (Burton et al., 1999), labelling multiple instances of that individual as the same person in card-sorting tasks (Jenkins et al., 2011; Zhou & Mondloch, 2016), and quicker to spot familiar individuals in arrays (Di Oleggio Castello et al., 2017; Dunn et al., 2018; Ito & Sakurai, 2014) than unfamiliar individuals. When matching unfamiliar face images, factors including whether the individuals are pictured wearing glasses (Kramer & Ritchie, 2016), sunglasses or masks (Noyes et al., 2019), the length of time between when the two images were taken (Megreya et al., 2013), and image colour (Bobak et al., 2019), can all impact accuracy and bias.

Ideally then, we should make judgements about familiar individuals when possible. However, CCTV operators, forensic investigators, passport officers, and cashiers are regularly required to make judgements about unfamiliar individuals’ identities. Studies have shown that even experienced passport officers perform at similar levels to the general population on an ID-card matching task (White et al., 2014), and that there were high rates of acceptance of fraudulent IDs in a study of supermarket cashiers (Kemp et al., 1997). It is important to note that there are certain individuals, dubbed super-recognisers (SRs), who are able to perform much more accurately in these tasks (Bobak et al., 2016a) than the general population. However, it is not always possible to have an SR present when an identification needs to be made. This, coupled with the knowledge that people generally have only moderate insight into their own face recognition abilities (Bobak et al., 2018), makes it valuable to develop other techniques of improving performance for face identification.

In practice, working in pairs (Dowsett & Burton, 2014) or larger groups (White et al., 2013) when making judgements about an unfamiliar person’s identity improves accuracy. With respect to the images themselves, showing idiosyncrasies such as open-mouthed smiles (Mileva & Burton, 2018) has been shown to enhance our ability to ‘tell faces together’ (Andrews et al., 2015; Burton et al., 2015). Another way of representing individual variability is to present multiple (Dowsett et al., 2016), highly variable images (Ritchie & Burton, 2016) of the target individual, as that leads to better learning of that individual. A recent study using a visual-search paradigm, showed that including four exemplars of a target individual led to improved accuracy in finding that individual in an array of distractors (Dunn et al., 2018). However, this study used cropped ‘floating’ heads for the task, and photographs were matched to static arrays of faces, which necessarily decreases ecological validity.

A previous study which used CCTV-like footage as stimuli showed reasonably high error rates (22% for target-present and 18% for target-absent conditions) when matching a live person to the video (Davis & Valentine, 2009). In a more recent experiment, SRs have been shown to be more accurate than controls in picking people from CCTV-footage of crowds (Davis et al., 2018). However, as stated above, finding SRs to perform video searches is not always possible. In a recent study of non SRs conducted by Kramer and colleagues (2020), they showed that in chokepoint videos, those where there is a narrowing of a passageway so as to allow only one person through at a time, performance for both target present and absent conditions was poor (~ 33%). When given three photographs of the target person, accuracy improved to approximately 46–57%, depending on how much variability there was between the photographs presented (Kramer et al., 2020). Due to their use of chokepoint footage, this task is more similar to a face-matching task, where you can look from the target image to the single face moving on screen, rather than a visual search task. Additionally, it may be that chokepoints are not always available as footage to use in these real-world visual search scenarios.

In real-life situations, such as the storming of the Capitol building, (USA, 2021), or the London riots (UK, 2011), videos and still images were compiled from CCTV and mobile-phone footage and someone in authority decided that these clips/stills were of the same identity. These decisions were most likely based on external factors like clothing and facial features, as they were taken on the same day. These were then released to the public or given to specialty task forces such as CCTV officers for identification of individuals within their own communities, where clothing and other external characteristics would differ. In other instances, such as for missing persons cases, or tracking criminal activity, CCTV officers are often given a single image of a target (personal communication with Glasgow CCTV operators) and asked to look for them as they scan live-streamed CCTV footage of the streets. This is similar to a visual search task.

In two experiments, we examined performance in a visual search task where targets were recorded in either CCTV-like (Experiment 1) footage from above, or body-camera/mobile-phone-like (Experiment 2) video footage from shoulder-height. In Experiment 1, participants were akin to the CCTV officers described above, where they studied and had access to either one target image or four, while viewing videos and deciding whether the target was present. Experiment 2 more closely resembles post-event analysis as body-camera and mobile-phone footage would, in most cases, not be ‘live-streamed’ and available immediately to an investigator. In both experiments we wanted to test whether the increased variability present within the four images would help with person identification. We predicted that viewing four target images of an individual prior to and during the visual search task would increase accuracy, in comparison to only having one image.

## Experiment 1

### Methods

#### Ethics

This experiment received ethical approval from the General University Ethics Panel at the University of Stirling.

#### Stimuli

We recruited 20 fourth year students at the University of Stirling (8 female) as targets in this experiment. All targets were white and in their early twenties. In order to emulate CCTV footage, targets were recorded at approximately 20 m, from above using an HD digital camera, while leaving a lecture theatre alongside other non-target individuals (target present). In addition, we recorded a series of videos of students leaving the lecture theatre where the targets did not appear (target absent). To match the density of the crowd, weather conditions, etc. the target absent footage was recorded immediately after the target present footage. There was no attempt to control the density of the crowd between target identities as we felt this would hinder the ecological validity of our experiment. All videos were cropped to 16 s in length. Each target also provided us with four images of them from social media, which included their face and body.

#### Participants

A total of forty students (28 female, Mage = 21.4, SDage = 8.1) from the University of Stirling were recruited to take part in this experiment, for course credit. All participants had normal or corrected-to-normal vision. A post hoc power analysis performed in JPower (Jamovi 1.2.27) indicated that with forty participants an alpha of 0.05 and 80% power, the minimum detectable effect size would be 0.45.

#### Design

This experiment was a within-subjects design with two independent variables, “target” (present/absent) and “number of exemplars” (1 or 4), and one dependent variable, accuracy in the visual search task.

#### Procedure

**Fig. 1:**
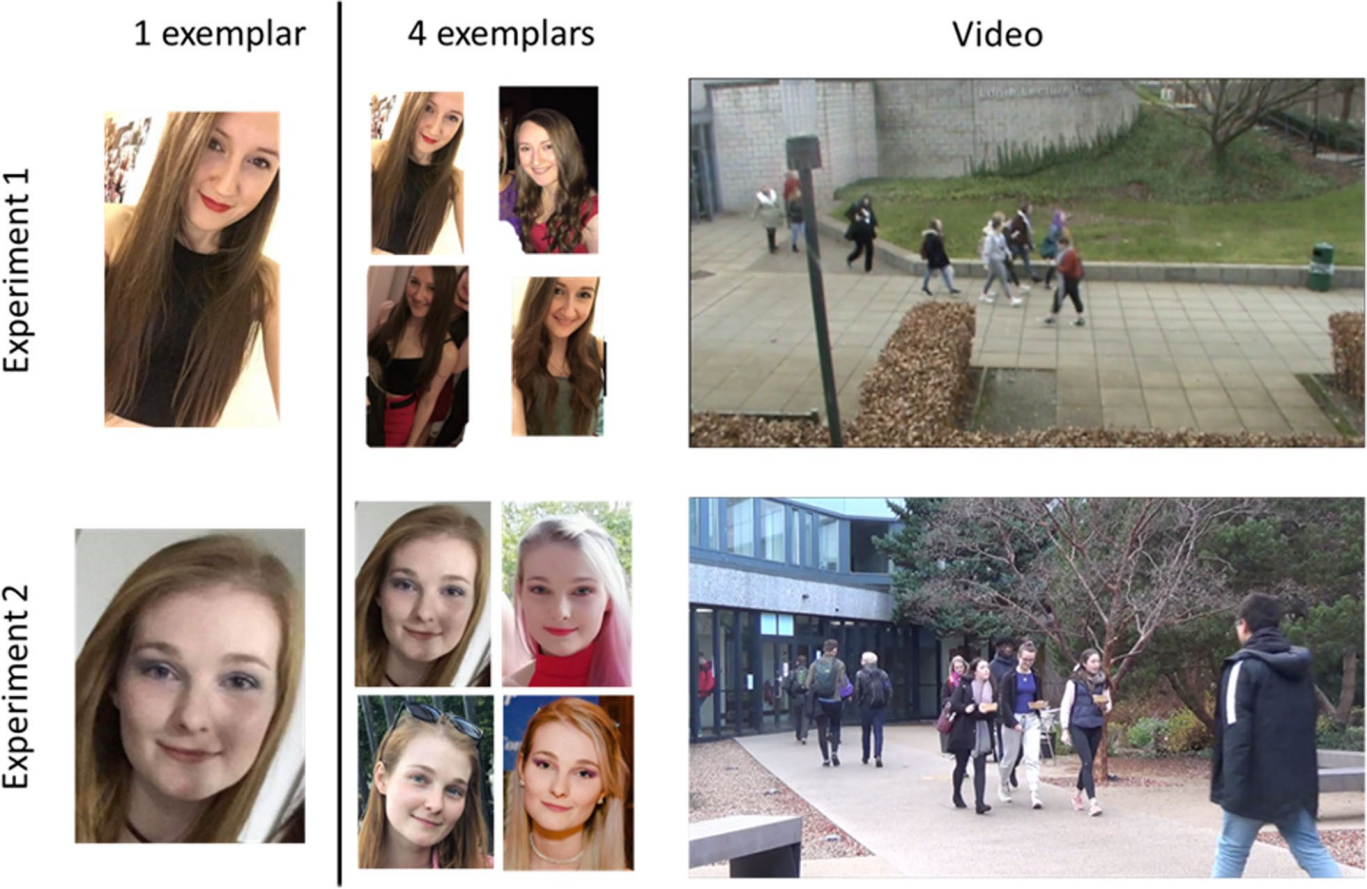
Example of a single trial in Experiment 1 (top row) and Experiment 2 (bottom row). Note that each target was seen in only one of the two exemplar conditions (1 vs. 4) for each participant

This experiment was set up in Eprime 2.0. Participants were seated approximately 60 cm away from a pair of computer monitors and were told that they would be participating in a visual search task. For each trial, on the left monitor appeared either a single image (1 exemplar) or four images (4 exemplars) of a target and the participant was asked to study these for 10 s. Subsequently, on the right monitor, participants were shown a 16 s CCTV-style video. Note that the images on the left monitor were displayed throughout the duration of the video on the right monitor so the participant could look back and forth between the two (Fig. 1, top row). This was done to simulate what a CCTV operator would typically be asked to do, when searching for a target individual through footage. Once the video was finished, both monitors displayed a white background and participants were asked whether the target they studied (left monitor) was present in the video they watched (right monitor), and to indicate their decision by pressing ‘Y’ for present or ‘N’ for absent.

There were 20 trials in total, with five videos for each of four conditions in this experiment: (1) 1 exemplar, target present; (2) 1 exemplar, target absent; (3) 4 exemplars, target present; (4) 4 exemplars, target absent. Across the experiment, each target was counterbalanced so that it appeared equally often in each of these conditions. In the 1 exemplar condition, the single image was randomly selected from the possible four. Finally, across trials, identities were never used as both stimuli and distractors.

### Results

We used JAMOVI (Jamovi.org, Version 1.2.27) for our data analyses, and all CIs reported for T-tests are for the effect size (Cohen’s d).

**Fig. 2:**
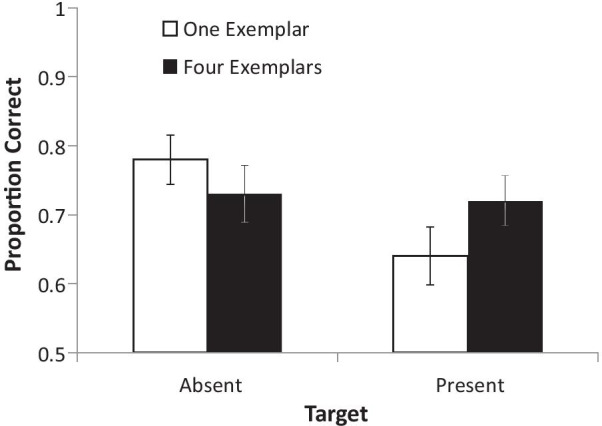
Accuracy (proportion correct) between target present and target absent conditions when participants viewed either 1 or 4 exemplars. Error bars show 95% CIs

Figure 2 shows the proportion correct in each condition. There appears to be little overall effect of number of exemplars but there is a suggestion of an interaction consistent with a more conservative decision threshold in the 1 exemplar condition: the hit rate is lower, and the false alarm rate is higher, in the 1 exemplar condition relative to the 4 exemplar condition.

To explore this, we performed signal detection analyses on these data. Sensitivity (d′) and bias (c) were calculated as per Bobak et al. (2016b). A paired-samples t-test revealed no difference in d’ between the 1 exemplar (*M* = 1.09, SE = 0.15) and 4 exemplar (*M* = 1.18, SE = 0.16) conditions, *t* (39) = 0.49, *p* = 0.63, *d* = 0.07, 95% CI on *d* [− 0.38, 0.23]. However, participants were marginally more likely to be conservatively biased, that is to decide a target was not in a video, when they only saw 1 exemplar (*M* = 0.19, SE = 0.07) than when they saw 4 exemplars (*M* = 0.02, SE = 0.06), *t*(39) = 1.88, *p* = 0.07, *d* = 0.30, 95% CI on *d* [− 0.02, 0.61].

### Discussion

These results suggest that there may be changes in participants’ bias depending on whether they are looking for a target individual in a video from a single image or a group of 4 images. Though not quite reaching statistical significance, participants appear to be more confident, or less conservative, when they have more exemplars to study.

In this experiment, the body of the individual being studied was also in view. Additionally, the video was shot from a great distance which could obscure facial features and lead to participants relying more heavily on body-feature cues to make their decisions. Indeed CCTV operators tend to rely on body-cues more heavily than facial-cues when making identity decisions from this distance (personal communication with Glasgow CCTV officers).

## Experiment 2

In Experiment 2, we sought to replicate and extend our findings in Experiment 1. In order to test face-matching ability and accuracy within a realistic visual-search task we cropped exemplar images so that they only included the face, shoulders, and hair. Additionally, we captured video footage that emulated what could be filmed by a mobile phone or a police body-camera. That is, unlike CCTV which tends to be filmed from a greater distance and above an individual of interest, this footage was filmed closer and at eye-level. We also increased the number of trials per participant within each condition.

### Methods

#### Stimuli

Thirty-two white third or fourth year undergraduate students (25 females), again in their early twenties, were recruited as stimuli for Experiment 2. We asked each person to walk through a busy courtyard at the University of Stirling while recording them using one of two HD cameras set up at the opposite entrances to the courtyard. Each target walked through the courtyard twice, once in each direction, with the clearest footage of each being used at test. Again, the footage recorded directly after the target was no longer visible on screen was used for target-absent trials. Each video was cropped to 30 s. Again, each target provided us with 4 images from social media which were then cropped to show only face, hair, and shoulders (Fig. 1, bottom row).

#### Participants

We recruited a total of 60 participants (42 women, Mage = 20.7, SDage = 3.4) in the same way as Experiment 1, to participate in this experiment. A post hoc power analysis performed in JPower (Jamovi 1.2.27) indicated that with sixty participants an alpha of 0.05 and 80% power, the minimum detectable effect size would be 0.37.

#### Procedure

Participants were asked to complete the Stirling Face Recognition Scale (SFRS) before performing the visual search task, for us to look at relationships between self-perceived face recognition ability and actual task performance. The task set-up was identical to Experiment 1; however, there were now 8 trials per condition with 32 trials in total, and each trial lasted for 30 s. Again, counterbalancing ensured that, across the experiment, each target appeared equally often in each of the experimental conditions.

### Results

**Fig. 3:**
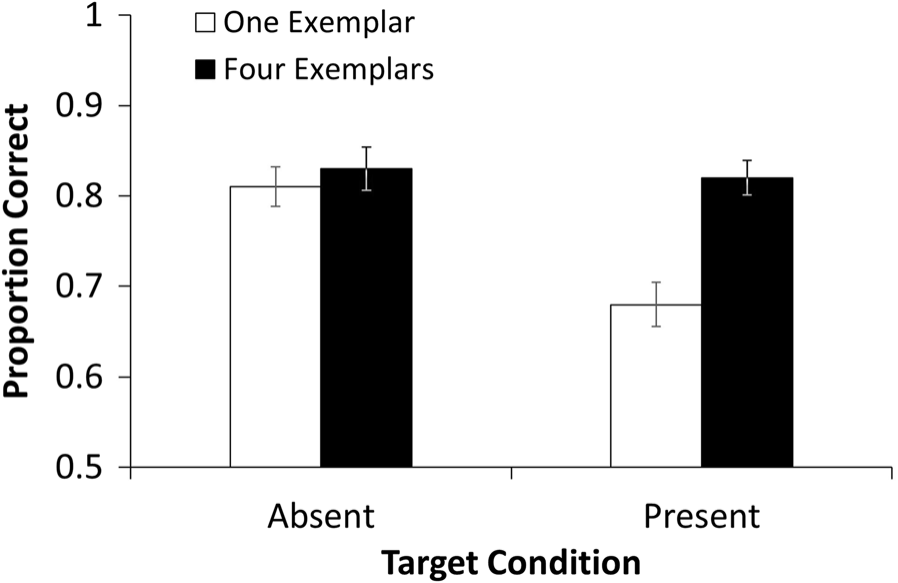
Accuracy (proportion correct) between target present and target absent conditions when participants viewed either 1 or 4 exemplars. Error bars show 95% CIs

Figure 3 shows accuracy for experiment 2. Performance appears to be better in the 4 exemplar condition, especially in target present trials.

We performed signal detection analysis as with Experiment 1. A paired-samples *t* test of *d*′ revealed that participants performed better in the task when they were able to study 4 exemplars (*M* = 1.94, SE = 0.08) rather than the 1 exemplar (*M* = 1.44, SE = 0.10), *t* (59) = 3.83, *p* < 0.001, *d* = 0.50, 95% CI on *d* [0.22, 0.76]. In addition, participants were significantly more likely to be conservatively biased, that is to decide a target was not in a video, when they only saw 1 exemplar (*M* = 0.21, SE = 0.05) than when they saw 4 exemplars (*M* = 0.03, SE = 0.05), *t*(59) = 2.50, *p* = 0.02, *d* = 0.32, 95% CI on *d* [0.06, 0.58].^1^

These results suggest that having 4 exemplars to look at before and during a visual search task leads to a higher hit rate than only having 1 exemplar, with no increase in false positives.

We were also interested in how well participant’s self-perceived face-recognition ability, measured using the SFRS, correlated with performance on this visual search task. In order to do this, we performed two correlations, one with the SFRS and 1 exemplar and one with SFRS and 4 exemplars. If the correlations with SFRS were positive, this would suggest that people with better self-perceived face-recognition abilities were better able to perform the search task. As it was, there was no significant correlation in the one exemplar task, *r* = 0.11, *p* = 0.41, 95% CI [− 0.15, 0.35], or in the four exemplar task, *r* = − 0.001, *p* = 0.99, 95% CI [− 0.26, 0.25]. These results suggest that either people do not have insight into their own face-recognition ability, as has been shown before (Bobak et al., 2018), or the SFRS may not tap into people’s ability to perform this particular task.

#### Item-analysis

As we only had 8 trials per condition, we performed a post hoc item-analysis to look at whether there were characteristics of specific targets which made them easier or harder for participants to identify (see Table 1 for descriptive statistics). Items were seen by fifteen participants in each condition. Firstly, signal detection analyses corroborated our findings that trials including 4 exemplars (*d*′: *M* = 2.13, SE = 0.18) were more discriminable than those with only 1 exemplar (*M* = 1.49, SE = 0.13), *t*(31) = 4.75, *p* < 0.001, *d* = 0.84, 95% CI on *d* [0.43, 1.23]. Additionally, trials with only 1 exemplar (*M* = 0.22, SE = 0.09) produced a more conservative bias than those with 4 exemplars (*M* = 0.01, SE = 0.08), *t*(31) = 3.48, *p* = 0.002, *d* = 0.61, 95% CI on *d* [0.23, 0.99].

**Table 1:** Means and ranges of stimuli items

Exemplars	Target present (hit rate)		Target absent (correct rejections)	
	1	4	1	4
Mean (SEM)	0.674 (0.04)	0.815 (0.03)	0.806 (0.02)	0.828 (0.03)
Minimum	0.130	0.330	0.270	0.330
Maximum	0.930	1.00	1.00	1.00

Additionally, we performed post hoc analyses to investigate how similar or variable the four exemplar images of each target were to each other, and whether this was correlated to performance on the four-exemplar task. We predicted that the more similar the four exemplars were, the lower the performance would be on the trials, as previous research shows that high variability images help with identification (Menon et al., 2015; Ritchie & Burton, 2016).

For this item-analysis we acquired ratings from 11 people in our laboratory (undergraduates, PhD’s, RAs, etc.) who were not familiar with the individuals presented. These participants were presented with 192 pairs of images (6 pairs × 32 targets). They were told that image pairs were of the same person but asked to ‘please decide how similar these two images look’ on a 6-point AFC scale with 1 being ‘extremely dissimilar’ and 6 being ‘extremely similar’. The average similarity between pairs of images for the 32 items was 4.15 (SE = 0.1), or approximately ‘rather similar’. There was a positive trend with similarity ratings and four-exemplar sensitivity, *r* = 0.30, *p* = 0.09, 95% CI [− 0.05, 0.59]. These results, although not formally significant, suggest that greater similarity between four images may lead to better performance, which was the opposite of our prediction.

**Fig. 4:**
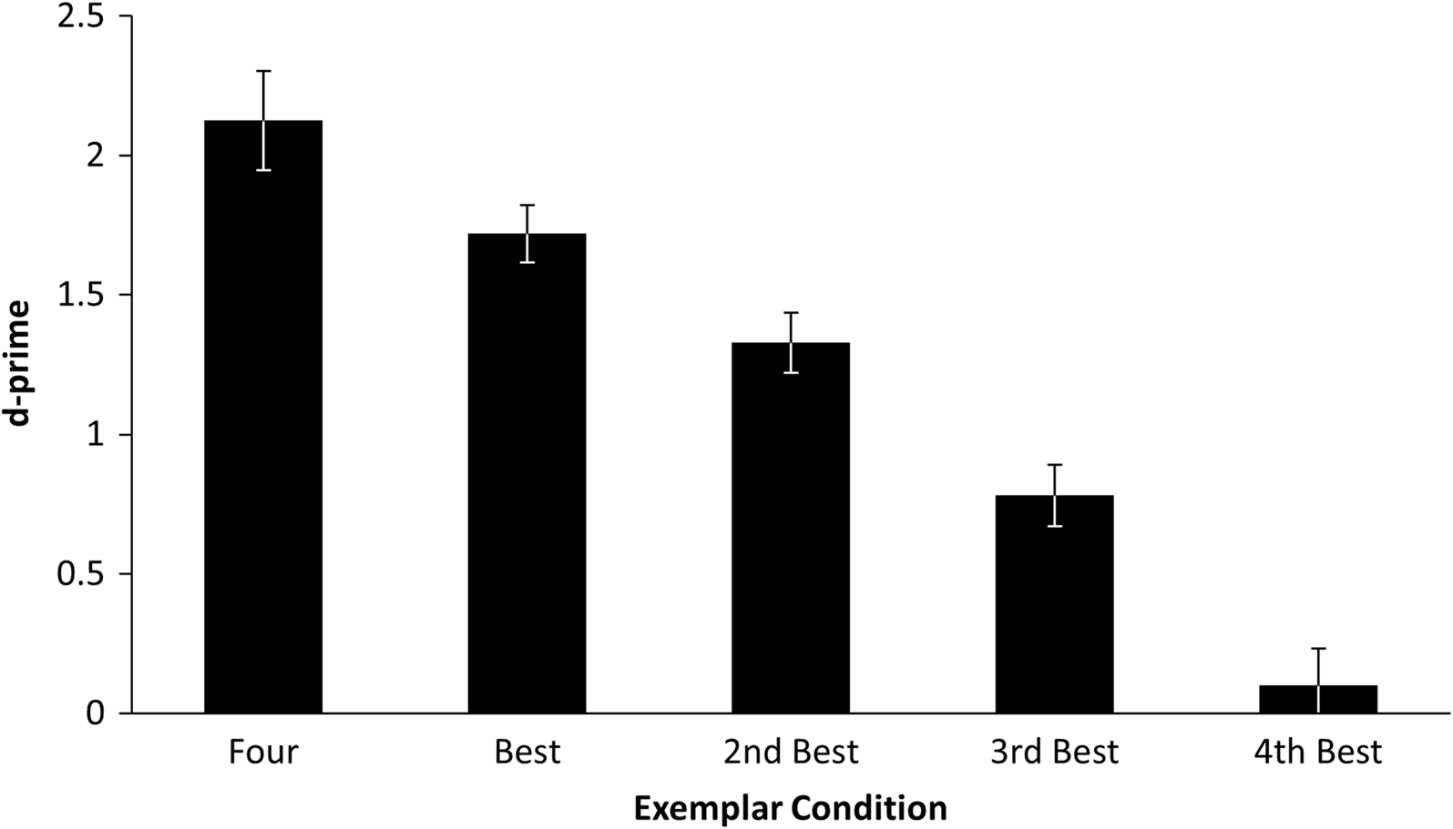
d-prime scores for the 4 exemplar condition and each of the exemplars for the 1 exemplar condition rank-ordered from best to worst. Error bars show SEM

Perhaps when participants have a choice between exemplars, they choose the ‘best’ one and use it to make their decision. If this were the case we would expect to see performance for the exemplar with the highest *d*′ in the 1 exemplar condition being similar to that of the *d*′ for the four exemplar condition. We calculated the *d*′ for each of the 4 exemplars of each target individual when they appeared in the 1 exemplar condition. Exemplars were then rank ordered from best to worst for each target identity. As individual exemplars in the 1 exemplar condition were selected at random from the set of four for each identity, two did not appear for any participants. Accordingly, all four exemplars from each of these two individuals were omitted from the analysis. This left a total of 120 exemplars (4 × 30 items). We adjusted the *d*′ calculation by adding 0.5 hits to those items where performance was at 0% and subtracting 0.5 where performance was at 100% as the function used to compute *d*′ is not defined at 0 or 1. The resulting average *d*′ scores in the four exemplar condition, and the best-to-worst scores the single exemplar condition are summarised in Fig. 4.

We performed a repeated measures ANOVA to examine whether there was any difference between the d’ for the 4 exemplar task and the best exemplar, 2nd best exemplar, 3rd best exemplar and 4th best in the 1 exemplar task. There was a main effect of Exemplar Condition, *F* (4, 116) = 89.90, *p* < 0.001, *η*^2^_*p*_ = 0.76 (Fig. 4). Follow-up Tukey tests revealed that there was a significant difference between each condition but the crucial comparison, 4 exemplars versus the best exemplar was highly significant *t*(116) = 3.42, *p* = 0.008. These results suggest that even though certain exemplars are better than others with respect to performance, the four-exemplar condition is still better than each individual one.

### Discussion

In Experiment 2 we cropped target images to include only the face and presented videos at approximately shoulder-height. We also recruited more participants than in Experiment 1. We found that participants were significantly more accurate in the target present conditions of the visual search task when presented with 4 exemplars of the target than when presented with only 1 exemplar. Participants performed more poorly and were more conservative in their decision making (more likely to decide a target was not in the video) when they were only given a single exemplar.

We also performed post hoc item-analyses which corroborated our sensitivity and bias findings for participants. However, we found no evidence that highly variable images led to better performance and instead found a non-significant trend that more similar images actually helped performance. We also found that participants were not simply using the best exemplar from the four, as performance was significantly better in the 4 exemplar condition than the best exemplar in the 1 exemplar condition.

## General discussion

For police officers, CCTV-operators, and security staff, finding unfamiliar individuals in video footage of crowds is often an unavoidable part of the job. They may be provided with images of a target person and asked to look through CCTV footage, mobile-phone footage, or through live CCTV feed to find the individual. In two experiments we examined performance in real-life visual search tasks where participants were given either 1 or 4 images of a target individual (see Fig. 1). In Experiment 1 participants were asked to look for the target individual in CCTV-like video footage, filmed from far away and above, while in Experiment 2, the footage was taken from closer and approximately shoulder height, similar to a mobile-phone recording. Across both experiments we found that in target present trials, four images improved accuracy compared with the one image condition, (from 64 to 72% in Experiment 1, and from 68 to 82% in Experiment 2); however, the result was only statistically significant in Experiment 2. Using signal detection analyses we found that in both experiments, participants were more conservative in their decision making when they were only given one target image. That is, they were more likely to decide that the target was not in the video when presented with only one image than when presented with four.

Many variables differed between Experiment 1 and Experiment 2. In addition to the placement of the camera for video capture, in Experiment 2 we also increased the number of trials and participants, which increased our ability to detect an effect. Images were also cropped so that a model’s face and hair were visible, but not their body. It is possible that participants relied on body cues for Experiment 1 (which is similar to CCTV operators—personal communication) and face cues for Experiment 2. For these reasons, which exact change led to the significant improvement seen in Experiment 2 is unclear. However, both studies are useful in identifying which contexts are used in applied visual search tasks (videos from high above or shoulder-height as well as full-body or face-only exemplars).

In a similar task to Experiment 2, only using chokepoint videos, Kramer et al. (2020) suggest that accuracy improves when participants view three images rather than one. However, their use of chokepoints inherently changes the task from a visual search to a matching task. In each video, a participant watched a single person enter or leave a lecture theatre, effectively being able to look back and forth between two individuals in order to make their decision. Additionally, participants were required to freeze the video and draw a box around the target, and then provide a confidence rating. This afforded participants more time to study both images, in effect choosing whether they were a match, as they could simply report low confidence if they did not think it was a plausible match (they were unable to pause and had to move onto the next trial once they had frozen the video in place). In our experiments, there were multiple people walking toward or away from the camera, directly in the visual search field, who needed to be excluded as potential targets.

Additionally, Kramer et al. (2020) found that supplying three images for participants to study was beneficial, but only when those images were of low variability (57% correct in low variability vs. 40% correct in high). Previous studies have shown that photographs of a target individual can vary greatly, and can be mistaken for several different individuals when the target is unfamiliar (Andrews et al., 2015; Jenkins et al., 2011). However, individuals vary in systematic ways (Burton et al., 2015) which can be learned and can improve performance in matching tasks (Mileva & Burton, 2018). Studying several highly variable images of an individual helps in learning and matching studies (Ritchie & Burton, 2016), which may be as a result of forming a more stable mental representation of how a specific individual varies between the images. Kramer et al. (2020) explain that the low variability images were taken at a similar time as their chokepoint videos, and as such could be more indicative of what the person looked like at the time the video was recorded. Our findings in Experiment 2, though not quite statistically significant, suggest that in trials where exemplars are more similar to each other, performance was better than trials where exemplars were rated as highly dissimilar. There may be some optimal level or type of variability that is useful in performing the task used in our experiments; that is, variability of the sort that one is likely to encounter in everyday life may improve performance. The images we used for our experiments were taken from targets’ social media and were mostly high variability images. These may not be indicative of the every-day differences one might expect of a student walking through a university campus. Indeed, many of the models provided images in which they had applied large amounts of cosmetics, or were selfies, both of which can drastically alter appearance.

Our data are consistent with the proposal from Ritchie et al. (2021) that multiple images are only helpful when there is a memory component to the task. They report that in simultaneous matching studies, providing multiple varied images does increase the correct matching rate but at the cost of also increasing false matches, resulting in no overall improvement in accuracy. If the task is sequential, so that participants need to remember what the target looks like, then there is an improvement. In our task, the presentation was simultaneous, in that participants could look back to the exemplars during the video, but there is clearly a memory component to the task as they had to search through the video for the target. The finding is consistent with the idea that multiple exemplars assist with forming a stable memory representation of a face (Ritchie & Burton, 2016), allowing better generalisation to a novel presentation of the individual depicted. Our item-analyses suggest that this is the case, as performance on the 4 template task was better than performance on any of the single exemplars in the 1 template task, including the highest performing one. Perhaps the 4 templates allow for a better stable representation of a face. However, this experiment was not set up explicitly to look at items, and further studies are needed.

In summary, providing several exemplars of a target individual of interest can help CCTV operators, police officers, and security guards in performing their visual search tasks more accurately; however, providing images with too much variability may cause performance to decrease. With the advent of social media and the abundance of personal images available online, providing additional images of missing persons or criminal suspects should be considered where possible.

## Data Availability

The datasets and analyses are available on the Open Science Framework (OSF) here: https://osf.io/264yd/.

## Footnotes

1 As we had a small number of trials, we examined whether our counterbalancing procedure had an effect on our results by including counterbalancing block as a between subjects factor. For *d*′ there was no interaction with counterbalancing block *F*(3, 56) = 0.48, *p* = 0.70, *η*_*p*_^2^ = 0.03, and no main effect, *F*(3, 56) = 1.45, *p* = 0.24, *η*_*p*_^2^ = 0.07. For criterion there was an interaction with counterbalancing block, *F*(3, 56) = 6.48, *p* < 0.001, *η*_*p*_^2^ = 0.26, but no main effect, *F*(3, 56) = 0.13, *p* = 0.94, *η*_*p*_^2^ = 0.01. We split the data by Counterbalancing block and performed paired-samples t-tests. Of the 4 blocks, 2 showed a significantly more conservative bias for the 1 template over the 4 template, *t*’s > 3.8, *p*’s < 0.002. The other 2 blocks, which vary slightly in the opposite direction did not reach significance, *t*’s < 1.05, *p*’s > 0.31.
